# Interleukin 6 promotes an *in vitro* mineral deposition by stem cells isolated from human exfoliated deciduous teeth

**DOI:** 10.1098/rsos.180864

**Published:** 2018-10-31

**Authors:** Nunthawan Nowwarote, Waleerat Sukarawan, Kiattipan Kanjana, Prasit Pavasant, Benjamin P. J. Fournier, Thanaphum Osathanon

**Affiliations:** 1 Excellence Center in Regenerative Dentistry, Faculty of Dentistry, Chulalongkorn University, Bangkok 10330, Thailand; 2 Department of Pediatric Dentistry, Faculty of Dentistry, Faculty of Dentistry, Chulalongkorn University, Bangkok 10330, Thailand; 3 Department of Anatomy, Faculty of Dentistry, Faculty of Dentistry, Chulalongkorn University, Bangkok 10330, Thailand; 4 Genomics and Precision Dentistry Research Group, Faculty of Dentistry, Chulalongkorn University, Bangkok 10330, Thailand; 5 Laboratory of Molecular Oral Pathophysiology, INSERM UMRS 1138, Cordeliers Research Center; Paris-Descartes; Pierre and Marie Curie; Paris, F-75006, France; Faculty of Dentistry, Paris Diderot University, Sorbonne Paris Cité, France

**Keywords:** interleukin 6, stem cells, deciduous teeth, osteogenic differentiation

## Abstract

Interleukin 6 (IL-6) plays various roles including stem cell regulation. The present study investigated the effect of IL-6 on cell proliferation, colony forming unit ability, stem cell marker expression and differentiation ability in stem cells isolated from human exfoliated deciduous teeth (SHEDs). We reported that the isolated cells from dental pulp tissues for deciduous teeth expressed CD44, CD90 and CD105 but not CD45. These cells were able to differentiate into osteoblasts, adipocytes and neuronal-like cells. IL-6 treatment resulted in the significant increase of *NANOG, SOX2* and *REX1* mRNA expression. However, IL-6 had no effect on cell proliferation and colony forming unit ability. IL-6 did not alter adipogenic and neurogenic differentiation potency. IL-6 supplementation in osteogenic medium led to a significant increase of mineralization. Furthermore, IL-6 upregulated *ALP, ANKH* and *PIT1* mRNA levels. In conclusion, IL-6 participates in the regulation of pluripotent marker expression and is also involved in mineralization process of SHEDs. Hence, IL-6 could be employed as a supplementary substance in culture medium to maintain stemness and to induce osteogenic induction in SHEDs for future regenerative cell therapy.

## 1. Introduction

Interleukin-6 (IL-6) is one of the IL-6-type cytokine family. IL-6 family includes IL-6, interleukin-11 (IL-11), ciliary neurotrophic factor (CNTF), cardiotrophin-1 (CT-1), leukaemia inhibitory factor, oncostatin and cardiotrophin-like cytokine (CLC). These molecules employ glycoprotein 130 (gp130) as a receptor to initiate signal transduction [1]. IL-6 is a biological cytokine function in immunity response, homeostasis, metabolism and cell development [2]. IL-6 promotes proliferation and multilayer formation of human epidermal keratinocytes *in vitro* [3]. Furthermore, IL-6 participates in osteoclast homeostasis via the regulation of receptor activator of nuclear factor *κ*B ligand (RANKL) and osteoprotegerin expression [4]. IL-6 directly inhibits RANK expression in osteoclast precursor cells, resulting in the inhibition of osteoclast formation [5]. This evidence indicates the multifunctional property of IL-6 in biological processes.

In cortical precursor cells, IL-6 binds to interleukin-6 receptor (IL-6R) and subsequently promotes self-renewal ability as well as maintaining cell numbers during embryogenesis [6]. IL-6 is highly expressed in human bone marrow-derived mesenchymal stem cells (MSCs). It enhances human MSCs proliferation but inhibits adipogenic and chondrogenic differentiation [7]. However, several contradictory studies indicate that IL-6 does not influence cell proliferation in human and mouse MSCs [8,9]. It has also been demonstrated that IL-6 and IL-6R supplementation results in the enhancement of chondrogenic differentiation of human MSCs as determined by the significant increase of pellet size and expression of chondrogenic marker genes (*COL2A1*, *ACAN* and *COL10A1*) [10]. This contradicted evidence requires further investigation to address the effect of IL-6 on stem cells' behaviours.

Stem cells isolated from human exfoliated deciduous teeth (SHEDs) were firstly described by Miura *et al.* [11]. Although, SHEDs exhibit mesenchymal stem cell characteristics, these cells exhibit distinct properties. In this regard, SHEDs have higher proliferation ability, but lesser osteogenic differentiation potency compared with human MSCs [11,12]. On the contrary, SHEDs showed better neurogenic differentiation potency [12]. This evidence suggested distinct phenotypes and properties of SHEDs. Previous studies have demonstrated that IL-6 participates in basic fibroblast growth factor (bFGF)-regulated REX1 expression in SHEDs [13]. However, the direct evidence regarding the influence of IL-6 on SHEDs stemness maintenance and multipotential differentiation remains lacking. The present study aimed to investigate the effect of IL-6 on SHEDs proliferation and differentiation ability toward osteogenic, adipogenic and neurogenic lineages.

## 2. Material and methods

### 2.1. Cell isolation and culture

Cell isolation procedure was approved by Human Research Ethic Committee, Faculty of Dentistry, Chulalongkorn University (Approval number 2017–096). Informed consent was obtained from parents. Deciduous teeth planned for extraction according to patient's treatment plan (e.g. shedding) were collected for cell isolation. Teeth that exhibited pathology (e.g. dental caries) were excluded. Briefly, teeth were rinsed with sterile normal saline and pulp tissues were gently removed in sterile condition. Pulp tissues were minced into small pieces and placed on 35 mm tissue culture dishes to allow cell migration out from the tissues. The explants cells were maintained in Dulbecco's Modified Eagle Medium (DMEM, Gibco, USA) supplemented with 10% fetal bovine serum (Hyclone, USA), 2 mM l-glutamine (Gibco, USA), 100 U ml^−1^ penicillin (Gibco, USA), 100 µg ml^−1^ streptomycin (Gibco, USA) and 5 µg ml^−1^ amphotericin B (Gibco, USA). The culture condition was maintained in 100% humidity, 37°C and 5% carbon dioxide. Culture medium was changed every 48 h. After reaching confluence, the cells were subcultured at 1 : 3 ratio. Cells at passage 3–7 were used in the experiments. In experimental groups, cells were treated with 10 ng ml^−1^ IL-6 (R&D System Inc, USA) [13].

### 2.2. Flow cytometry analysis

Cells were detached with trypsin/EDTA solution to obtain single-cell suspension. Further, cells were washed with 1% FBS in PBS and subsequently stained with antibodies. Primary antibodies were FITC conjugated anti-human CD44 (BD Bioscience Pharmingen, USA), APC-conjugated anti-human CD90 (Immuno Tools, Germany), PE-conjugated anti-human CD105 (Immuno Tools) and PerCP-conjugated anti-CD45 (Immuno Tools). Stained cells were analysed using a FASCalibur using the CellQuest software (BD Bioscience, USA).

### 2.3. Proliferation and colony forming unit assay

MTT assay was employed for cell proliferation evaluation. Briefly, cells were seeded in 24-well plates at density of 12 500 cells per well. At designated time points, cells were incubated with 1 mg ml^−1^ 3-(4,5-dimethylthiazol-2-yl)-2,5-diphenyltetrazolium bromide solution for 15 min at 37°C to allow precipitation of formazan crystals. The formazan crystals were solubilized in dimethyl sulfoxide-glycine buffer and the absorbance was examined at 570 nm.

For colony forming unit assay, 500 cells were plated on 60 mm tissue culture dishes and maintained in growth medium. Culture medium was changed every 48 h. At day 14, cells were washed with sterile PBS and fixed with 4% paraformaldehyde solution for 10 min. Colony formation was visualized by staining with Coomassie Blue (Sigma, USA). The percentage of colony area was analysed using ImageJ software.

### 2.4. Differentiation induction

Differentiation protocols were performed using methods described in previous publications [13,14]. Osteogenic differentiation was induced by incubating cells with osteogenic induction medium (OM; growth medium supplemented with 50 µg ml^−1^ ascorbic acid, 10 mM β-glycerophosphate and 100 nM dexamethasone). Medium was changed every 48 h. Mineral deposition was evaluated using Alizarin Red S staining. Briefly, samples were fixed with cold methanol for 10 min, washed with deionized water, and further incubated with 1% Alizarin Red S solution (Sigma, USA) for 3 min at room temperature under gentle agitation. Excess staining was washed by deionized water. The staining was eluted in cetylpyridinium chloride solution and the absorbance was measured at 570 nm. Osteogenic marker gene expression was determined using real-time polymerase chain reaction.

For adipogenic differentiation, cells were maintained in growth medium supplemented with 0.1 mg ml^−1^ insulin, 1 mM dexamethasone, 1 mM IBMX and 0.2 mM indomethacin. Oil Red O staining was performed to examine intracellular lipid accumulation at day 16 after induction. Briefly, samples were fixed with 4% paraformaldehyde for 30 min at room temperature and subsequently incubated with 60% isopropanol for 5 min. Samples were then stained with Oil Red O solution for 5 min at room temperature. Adipogenic marker gene expression was determined using real-time polymerase chain reaction.

For neurogenic induction, neurosphere formation assay was performed by seeding cells on Petri dishes. Cells were maintained in neurobasal medium containing 2% B27, 2 mM l-glutamine, 100 U ml^−1^ penicillin, 100 µg ml^−1^ streptomycin, 5 µg ml^−1^ amphotericin B, 20 ng ml^−1^ bFGF and 20 ng ml^−1^ EGF. At day 7 after induction, neurospheres were fixed with 4% paraformaldehyde solution and the protein expression of neurogenic marker (*β*3-Tubulin) was examined using immunofluorescence staining. Neurogenic marker gene expression was determined using real-time polymerase chain reaction.

### 2.5. Immunofluorescence staining

*β*3-Tubulin expression was evaluated using immunofluorescence staining. Neurospheres were fixed with 4% paraformaldehyde solution for 10 min and permeabilized using 0.1% Triton^®^-X100 at room temperature. Unspecific antibody binding was blocked by incubating with 10% horse serum in PBS for 1 h. Subsequently, neurospheres were incubated with primary antibody (anti *β*3-Tubulin 1 : 100 dilutions, Promega, USA) for 18 h at room temperature. Biotinylated-goat anti-mouse antibody (Invitrogen, USA) was employed as secondary antibody (1 : 500 dilution). Samples were incubated with secondary antibody for 1 h at room temperature. Streptavidin-FITC (Sigma, USA) was incubated for 30 min for the detection of protein expression. Samples were gently washed three times with phosphate-buffered saline between each step. Nuclei were counterstained with DAPI. The omitting of primary antibody protocol was employed as the negative control. The expression of *β*3-Tubulin was observed under fluorescence microscope (Axiovert 40 CFL; Carl Zeiss, Germany).

### 2.6. Polymerase chain reaction

RiboEx total RNA isolation solution (GeneAll, Seoul, South Korea) was used for total RNA extraction. The integrity and amount of RNA was evaluated using Nanodrop equipment. One microgram of total RNA was converted to complementary DNA using reverse transcriptase ImPromII kit (Promega, Madison, WI, USA). cDNA (1 µl) was subjected to real-time polymerase chain reaction using a FastStart Essential DNA Green Master kit (Roche Diagnostic, USA). The reaction was performed on MiniOpticon real-time PCR system (Bio-Rad, USA). Target gene expression value was normalized to *18S* expression value and subsequently normalized to the control. Primer sequences are shown in electronic supplementary material, table S1.

### 2.7. Statistical analyses

Data were presented as box and whisker plot. The upper and lower bar indicated the maximum and minimum values, respectively. The upper and lower borders of the box indicated 3rd and 1st quartile, respectively. The median value was indicated as the line in the box. All data points were demonstrated as black dots in the plot. Cells from at least four different donors were employed in each experiment. Statistical analyses were performed using Prism 7 (GraphPad Software, CA, USA). For two independent group comparison, Mann–Whitney *U*-test was employed. For three or more group comparison, Kruskal–Wallis test was performed followed by pairwise comparison with Type I error correction. The statistical significance was considered at *p* < 0.05.

## 3. Results

### 3.1. Characterization of stem cell characteristics

**Figure 1.**
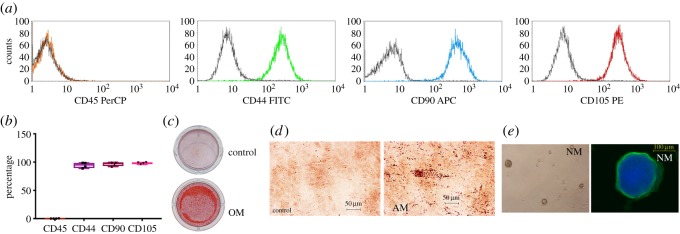
Characterization of stem cell characteristics. Expression of surface markers was evaluated using flow cytometry analysis (*a,b*). Cells were maintained in OM for 14 days and the mineral deposition was examined using Alizarin Red S staining (*c*). For adipogenic differentiation, cells were cultured in AM for 16 days and the intracellular lipid accumulation was observed using Oil Red O staining (*d*). For neurogenic induction, cells were cultured with neurogenic medium (NM) in Petri dishes for 7 days. *β*3-Tubulin expression was evaluated using immunofluorescence staining (*e*).

The isolated cells were characterized for mesenchymal stem cell characteristics. Results showed that cells did not express a hematopoietic stem cell marker, CD45 (0.12 ± 0.21%) but exhibited the positive staining for mesenchymal stem cell markers, CD44 (94.33 ± 4.95%), CD90 (96.76 ± 3.63%) and CD105 (98.03 ± 1.66%) (figure 1*a,b*). After maintaining cells in OM for 14 days, the marked increase of mineral deposition was observed compared with those cells cultured in growth medium (figure 1*c*). Further, intracellular lipid accumulation was observed in those cells maintained in adipogenic induction medium (AM) at day 16 (figure 1*d*). When cells were seeded on Petri dishes and maintained in neurogenic induction medium (NM) for 7 days. The neurospheres were formed and these spheres expressed a neurogenic marker: *β*3-Tubulin (figure 1*e*).

### 3.2. IL-6 enhanced pluripotent stem cell marker expression

**Figure 2.**
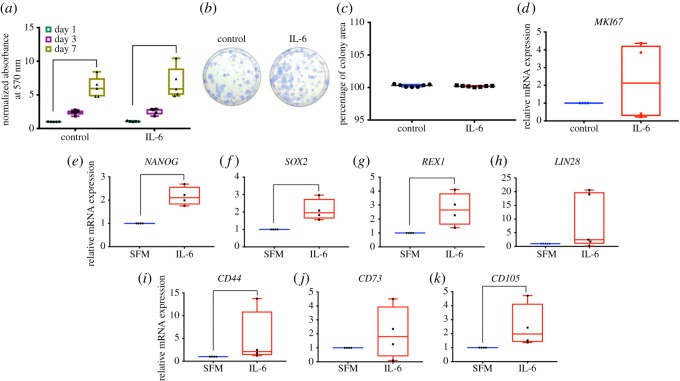
Effect of IL-6 on stemness properties. Cells were maintained in growth medium supplemented with or without IL-6 (10 ng ml^−1^). Cell proliferation was determined using MTT assay. Graph demonstrated the normalized absorbance at days 1, 3 and 7 (*a*). Colony forming unit was observed using Coomassie Blue staining at day 14 (*b*). Graph represented the percentage of colony area determined by ImageJ software (*c*). The mRNA expression of *MKI67* was evaluated using real-time polymerase chain reaction (*d*). For stem cell marker expression, cells were maintained in SFM with or without IL-6 supplementation for 24 h. The relative mRNA levels were determined by real-time polymerase chain reaction (*e*–*k*). The bars indicate a significant difference (*p* < 0.05).

Cells were maintained in growth medium supplemented with or without IL-6 (10 ng ml^−1^). At 1, 3 and 7 days after culture, cell proliferation was determined using MTT assay. Results demonstrated that cells were able to proliferate *in vitro* as the significant difference was observed at day 7 compared with day 1. An addition of IL-6 did not influence cell proliferation ability as determined by MTT assay and methylene blue staining (figure 2*a* and electronic supplementary material, figure S1, respectively). Moreover, IL-6 did not affect the colony forming unit ability (figure 2*b*). The percentage of colony area was comparable between cells in the control condition and those supplemented with IL-6 (figure 2*c*). Correspondingly, the expression of proliferative marker, *MKI67*, was not significantly different after treating cells with IL-6 for 24 h (figure 2*d*).

To evaluate the influence of IL-6 on stem cell marker expression, cells were maintained in serum free medium (SFM) and treated with IL-6 for 24 h. IL-6 treated cells expressed significant higher pluripotent stem cell markers; *NANOG*, *SOX2* and *REX1* (figure 2*e*–*g*). *LIN28* mRNA levels seemed upregulated in IL-6 treated group; however, no significant difference was observed (figure 2*h*). Furthermore, *CD44* and *CD105* mRNA expressions were upregulated in IL-6 treated groups while no significant change was observed for *CD73* mRNA levels (figure 2*i*–*k*). However, there was no significantly difference of CD44 and CD105 protein expression between IL-6 treated cells and the control at 48 h (electronic supplementary material, figure S2).

### 3.3. IL-6 has no influence on adipogenic and neurogenic differentiation of SHEDs

**Figure 3.**
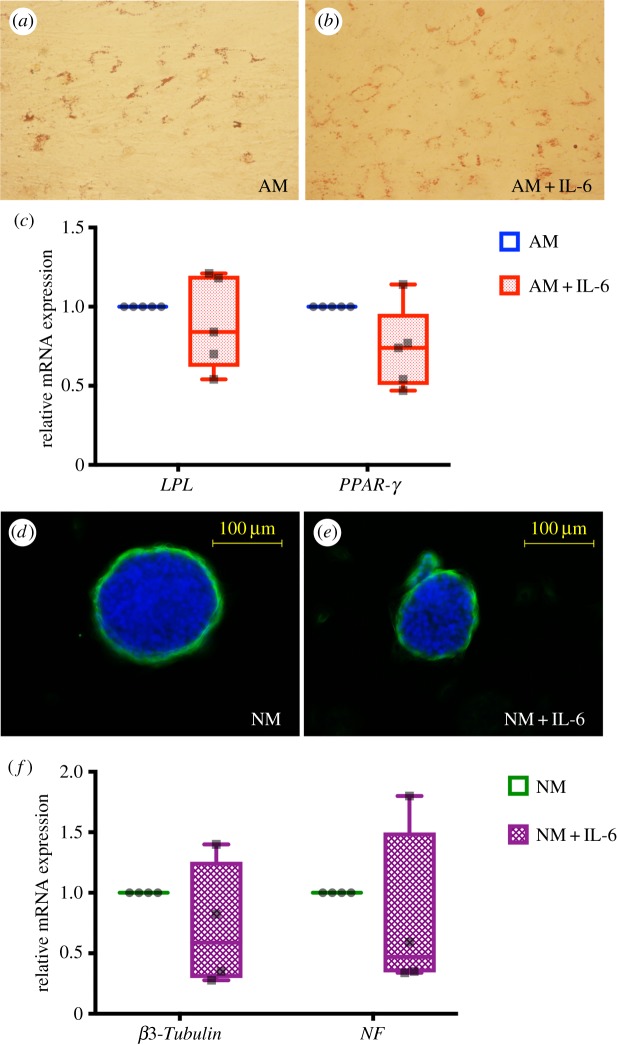
Effect of IL-6 on adipogenic and neurogenic differentiation in SHEDs. Cells were cultured in AM in the presence or the absence of IL-6 (10 ng ml^−1^). An intracellular lipid accumulation was examined using Oil Red O staining at day 16 (*a,b*). Adipogenic marker gene expression was determined using real-time polymerase chain reaction at day 8 after induction (*c*). Cells were maintained in neurogenic induction medium (NM) in the presence or the absence of IL-6 (10 ng ml^−1^) and cultured in Petri dishes for 7 days. *β*3-Tubulin expression was determined using immunocytochemistry staining (*d,e*). The mRNA levels of neurogenic markers were observed using real-time polymerase chain reaction (*f*).

SHEDs were maintained in AM in the presence or the absence of IL-6 (10 ng ml^−1^). At day 16, results demonstrated that there was no marked difference of intracellular lipid accumulation as determined by Oil Red O staining (figure 3*a,b*). Correspondingly, the mRNA levels of *LPL* and *PPAR-γ* were not significantly different between the control (AM) and IL-6 treated groups (AM + IL-6) (figure 3*c*).

After neurosphere formation for 7 days, the spheres in both the control (NM) and IL-6 treated groups (NM + IL-6) exhibited similar size and morphology. In addition, the *β*3-Tubulin levels were not different between both groups (figure 3*d,e*). The mRNA expression of *β3-Tubulin* and *NF* was also not altered in IL-6 treated groups (figure 3*f*).

### 3.4. IL-6 upregulated mineralization and genes related to phosphate metabolism in SHEDs

**Figure 4.**
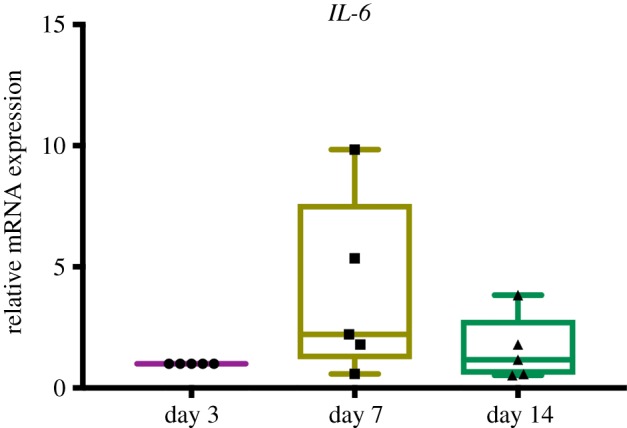
Endogenous *IL-6* expression during osteogenic differentiation in SHEDs. Cells were maintained in OM for 3, 7 and 14 days. Total RNA was collected and the mRNA expression of *IL-6* was determined using real-time polymerase chain reaction.

To determine role of endogenous *IL-6* on osteogenic differentiation in SHEDs, the *IL-6* mRNA expression pattern during osteogenic induction period was evaluated. In normal osteogenic induction, SHEDs exhibited an increase trend of *IL-6* mRNA expression at day 7 compared with day 3. However, there is no statistically significant difference (figure 4).

**Figure 5.**
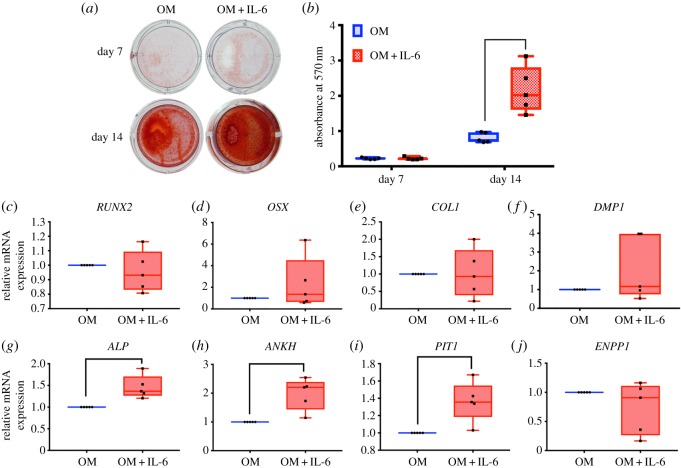
IL-6 on osteogenic differentiation in SHEDs. Cells were maintained in OM in the presence or the absence of IL-6 (10 ng ml^−1^). At days 7 and 14, mineral deposition was determined by Alizarin Red S staining (*a*). Graph demonstrated the absorbance of solubilized dye solution at 570 nm (*b*). The expression of osteogenic marker and phosphate regulatory genes was evaluated by real-time polymerase chain reaction at day 7 after osteogenic induction (*c*–*j*). The bars indicate a significant difference (*p* < 0.05).

SHEDs were cultured in OM supplemented with IL-6 (10 ng ml^−1^). Mineral deposition was examined using Alizarin Red S staining at day 7 and 14 (figure 5*a,b*). Results illustrated that IL-6 significantly promoted mineralization in SHEDs at day 14 compared to control (OM). There was no significant difference of osteogenic marker gene (*RUNX2, OSX, COL1* and *DMP1*) expression between the control and IL-6-treated condition (figure 5*c*–*f*).

Furthermore, genes involved in mineralization process were evaluated. IL-6 significantly upregulated *ALP, ANKH* and *PIT1* mRNA expression (figure 5*g*–*i*). The mRNA levels of *ENPP1* were not significantly different in IL-6-treated conditions (figure 5*j*).

## 4. Discussion

In the present study, we investigated the effect of IL-6 on pluripotent stem cell markers expression and differentiation potency of SHEDs. The results showed that IL-6 significantly enhanced stem cell markers expression; however, there was no influence on cell proliferation and colony forming unit ability. Furthermore, the addition of IL-6 in OM significantly promoted mineralization. However, IL-6 did not change adipogenic and neurogenic differentiation abilities.

The present study described the characterization of cells isolated from remaining dental pulp tissues of shedding deciduous teeth. The results suggested that these isolated cells exhibited the characteristics of MSCs. *In vivo*, stem cells maintain their stemness state by the regulation of residence microenvironment, the so-called niche. The cultivation of stem cells *in vitro* is considered as a separate system where only stem cells are cultured in the tissue culture plate. Hence, the stemness regulation from niche is lacking. The *in vitro* long-term culture of stem cells may lead to spontaneous cell differentiation. Previous study from our group demonstrated that SHEDs at passage 10 exhibited reduced proliferation ability and loss of pluripotent stem cell marker expression [15]. In order to maintain stemness state of stem cells *in vitro*, chemical agents and growth factors are added in the culture medium to mimic the regulation occurring in niche. The present study demonstrated that IL-6 supplementation in culture medium enhanced pluripotent stem cell marker expression, suggesting that IL-6 may be one of the molecules in niche of SHEDs and regulates their stemness maintenance.

IL-6 significantly upregulated pluripotent stem cell markers, *NANOG, SOX2* and *REX-1* in serum free conditions. Previous works from our group reported that mechanical stress regulated pluripotent stem cell marker, *REX-1*, in SHEDs via IL-6 [16]. In this regard, neutralizing antibody against IL-6 and IL-6 siRNA treatment decreased the mechanical stress-induced *REX-1* expression. IL-6 treatment induced REX-1 expression via ATP/P2Y1 pathway [17]. Moreover, IL-6 also participated in bFGF-induced *REX-1* expression in SHEDs as the neutralizing antibody against IL-6 reduced bFGF-induced REX-1 expression [13]. The present study also demonstrated that IL-6 significantly induced mesenchymal stem cell marker, *CD44* and *CD105*, expression. Correspondingly, previous report showed that IL-6 treatment enhanced CD44 (variant 6) expression in cancer cells via STAT3 activation [18,19]. However, the present study demonstrated that the protein expression of CD44 and CD105 was not significantly different between IL-6 treated cells and the control, suggesting the different regulation of IL-6 on the transcription and translation process of these molecules. The regulation of translational process of IL-6 on CD44 and CD105 should be further examined. The contradicted results of IL-6 regulated CD44 and CD105 gene and protein expression should be further elucidated. Taken together, IL-6 may play a crucial role in stemness maintenance in SHEDs and may participate in various regulatory mechanisms.

IL-6 regulated cell proliferation has been reported in various cell types. The present study demonstrated that IL-6 had no influence on SHEDs proliferation and colony forming unit. Similarly, previous reports demonstrated that IL-6 had no effect on cell proliferation in human adipose-derived MSC and mouse bone marrow-derived MSC [8,20]. However, it has been shown that IL-6 enhanced cell proliferation in human placental MSC and bone marrow-derived MSCs [7,21]. Correspondingly, an inhibition of endogenous *IL-6* expression resulted in the reduction of cell proliferation ability in bone marrow-derived MSC [7]. These discrepancies could be due to the different cell sources. Different cell types may express different levels of IL-6R which may influence the different cell responses. In addition, these studies employed various IL-6 concentrations, which may be reflected from different IL-6R levels. The mechanisms, by which IL-6 upregulated pluripotent marker expression but did not affect cell proliferation in specific cell types, should be further elucidated.

IL-6 participates in the regulation of stem cell differentiation. The present study showed that IL-6 had no influence on adipogenic ability in SHEDs. Similarly, IL-6 supplementation in AM did not influence intracellular lipid accumulation and adipogenic marker gene expression in murine stromal vascular cells, human bone marrow-derived MSCs and human adipose-derived MSCs [20,22,23]. On the contrary, an addition or pre-treatment of exogenous IL-6 led to the reduction of adipogenic differentiation ability as determined by the decrease of intracellular lipid accumulation and adipogenic marker gene expression, corresponding with the reduction of endogenous *IL-6* mRNA expression upon adipogenic differentiation [7]. Hence, the regulation of adipogenic differentiation by IL-6 in specific stem cell types should be further investigated.

The combination of IL-6 and sIL-6R has been shown to promote neuronal differentiation in human-induced pluripotent stem cell-derived neuronal stem cells via the STAT3 pathway [24]. However, the present study illustrated that addition of IL-6 in neurogenic induction medium had no effect on neurogenic marker expression and neurosphere formation. This discrepancy could be due to the participation of sIL-6R. It has been shown that IL-6 alone did not influence neurogenic differentiation in mouse neuronal stem cells [25]. However, IL-6/sIL-6R complex significantly promoted the expression of neurogenic markers in both mRNA and protein levels [25]. Thus, an impact of IL-6/sIL-6R complex on neurogenic differentiation ability in SHEDs should be further examined.

The significant increase of IL-6 was observed during osteogenic differentiation of human bone marrow-derived MSC in both mRNA and protein levels [26], suggesting the role of endogeneous IL-6 during osteogenic differentation. However, the present study found the trend of increasing *IL-6* mRNA expression during osteogenic differentiation but there was no statistically significant difference. Hence, the endogenous IL-6 may not involve in osteogenic differentiation in SHEDs. On the contrary, the present study illustrated that an exogeneous IL-6 enhanced mineralization and phosphate metabolism-related gene expression in SHEDs under osteogenic induction culture. In OM, dexamethasone supplementation promotes osteogenic differentiation while β-glycerophosphate can be cleaved by ALP enzyme releasing from differentiating osteogenic cells into inorganic phosphate and subsequently contributes in calcium phosphate crystal precipitation. When the OM was supplemented with IL-6, the significant increase of mineralization was observed at day 14, suggesting the additional influence of IL-6 on SHEDs mineralization under osteogenic induction condition. The significant change of genes related to phosphate metabolism was also demonstrated, confirming the contribution of IL-6 in osteogenic induction condition.

Our results also showed that an addition of IL-6 in OM resulted in the significant increase of *ALP* expression and mineralization *in vitro.* Similar to the study in human bone marrow-derived MSC, exogenous IL-6 promoted mineralization while IL-6 antibody inhibited an *in vitro* mineral deposition [26]. This regulation was potentially regulated by the phosphoSTAT3 pathway [26]. Furthermore, IL-6 injection promoted new bone formation in BMP-2 induced ectopic bone formation in rats [22]. However, it has been reported that IL-6 had no effect on osteogenic differentiation potency as evaluated by mineralization ability and ALP activity in human bone marrow-derived MSC [7]. The difference between these studies is the concentration of IL-6 used in the experiment. High IL-6 concentration (100 ng ml^−1^) promoted osteogenic differentiation in human bone marrow-derived MSC while low IL-6 concentration (10 ng ml^−1^) did not have an effect. The present study demonstrated that low IL-6 concentration (10 ng ml^−1^) was able to enhance mineralization ability in SHEDs.

The increased expression of genes involved in phosphate metabolism has been proposed as the mechanism by which IL-6 enhanced mineral deposition in SHEDs upon osteogenic differentiation. In this regard, the significant upregulation of *ALP*, *ANHK* and *PIT1* mRNA expression was observed in IL-6 treated SHEDs. Similar to previous report, IL-6 enhanced crystal deposition *in vitro* and upregulated *Ank* and *Pit1* mRNA expression in primary mouse chondrocytes [27]. ANK transports pyrophosphate from intracellular to extracellular site, resulting in the accumulation of extracellular pyrophosphate. Accompanied by the increase of ALP, extracellular pyrophosphate could be cleaved by ALP into inorganic phosphate, and these inorganic phosphate ions can be transported into intracellular site by the function of PIT1. These mechanisms may lead to the induction of osteogenic differentiation and mineralization [28,29].

The present study employed cells from at least four different donors in each experiment to demonstrate the biological replications. Indeed, cells from different donors exhibited different baseline of cell behaviours (e.g. gene expression) due to biological variation among population. Hence, the present study performed the normalization of the experimental value against the control of each donor to determine fold difference so that the trend of IL-6 effect can be compared among donors. Since there are relative values, the interpretation should be done with caution. However, the effect of IL-6 in SHEDs can be clearly demonstrated in mineralization assay, as IL-6 treated SHEDs exhibited significant higher mineral deposition than the control, suggesting the change of cell function in IL-6 treated condition.

## 5. Conclusion

The present study showed that IL-6 is a cytokine regulating stem cell function in SHEDs. Exogenous IL-6 enhanced pluripotent stem cell markers in SFM and also induced mineral deposition in osteogenic induction conditions by upregulating genes related to phosphate metabolism in SHEDs. These finding could be a useful approach to control stem cell lineage in future regenerative treatments.

## Supplementary Material

Supplementary Table 1

## Supplementary Material

Supplementary Figure 1

## Supplementary Material

Supplementary Figure 2

## Data accessibility

Additional information is available in the electronic supplementary material.
