# Supplementary material for: Interleukin 6 promotes an in vitro mineral deposition by stem cells isolated from human exfoliated deciduous teeth
Source: R Soc Open Sci. 2018 Oct 31;5(10):180864. doi: 10.1098/rsos.180864 (PMC6227976; doi:10.1098/rsos.180864)
Supplement: Supplementary Table 1 [file rsos180864supp1.docx]

**Supplementary Table 1.** Primer sequences.

**Gene Accession Number Primer sequences**

*IL-6*  NM000600.4 (Forward) 5’ ATGCAATAACCACCCCTGAC 3’

(Reverse) 5’ AAAGCTGCGCAGAATGAGAT 3’

*MKI67* NM_001145966.1 (Forward) 5′ AGAAGAAGTGGTGCTTCGGAA 3'

(Reverse) 5′ AGTTTGCGTGGCCTGTACTAA 3′

*REX1* NM174900.4 (Forward) 5’ TGGGAAAGCGTTCGTTGAGA 3’ (Reverse) 5’ CACCCTTCAAAAGTGCACCG 3’

*SOX2* NM003106.3 (Forward) 5’ ACCAGCTCGCAGACCTACAT 3’

(Reverse) 5’ ATGTGTGAGAGGGGCAGTGT 3’

*NANOG* NM024865.3 (Forward) 5’ATGCCTCACACGGAGACTGT 3’

(Reverse) 5’AAGTGGGTTGTTTGCCTTTG 3’

*LIN28A* NM024674.5 (Forward) 5’ GAGCATGCAGAAGCGCAGATCAA 3’

(Reverse) 5’ TATGGCTGATGCTCTGGCAGAAG 3’

*CD44* NM001202557.1 (Forward) 5’ ACAAGTTTTGGGGGCACGCA 3’

(Reverse) 5’ CAATCTTCTTCAGGTGGAGC 3’

*CD73* NM002526.2 (Forward) 5’ ACACTTGGCCAGTAAAATAGGG 3’

(Reverse) 5’ ATTGCAAAGTGGTTCAAAGTCA 3’

*CD105* NM001114753.1 (Forward) 5’ CATCACCTTTGGTGCCTTCC 3’

(Reverse) 5’ CTATGCCATGCTGCTGGTGGA 3’

*ALP*  NM000478.5 (Forward) 5’ GACCTCCTCGGAAGACACTC 3’

(Reverse) 5’ TGAAGGGCTTCTTGTCTGTG 3’

*COL1* NM000088.3 (Forward) 5’ GTGCTAAAGGTGCCAATGGT 3’

(Reverse) 5’ ACCAGGTTCACCGCTGTTAC 3’

*DMP1* NM 004407.3 (Forward) 5’ CAGGAGCACAGGAAAAGGAG 3’

(Reverse) 5’ CTGGTGGTATCTTGGGCACT 3’

*RUNX2* NM001024630.3 (Forward) 5’ ATGATGACACTGCCACCTCTG 3’

(Reverse) 5’ GGCTGGATAGTGCATTCGTG 3’

*OSX* NM001173467.2 (Forward) 5' GCCAGAAGCTGTGAAACCTC 3'

(Reverse) 5’ GCTGCAAGCTCTCCATAA 3’

*LPL*  NM000237.2 (Forward) 5’ GAGATTTCTCTGTATGGCACC 3'

(Reverse) 5' CTGCAAATGAGACACTTTCTC 3'

*PPARγ*  NM138712.3 (Forward) 5′CCAGTGGTTGCAGATTACAAGTATG 3′

(Reverse) 5′TTGTAGAGCTGAGTCTTCTCAGAATAATAAG 3'

*NF* NM006158.4 (Forward) **5' ACCCGACTCAGTTTCACCAG 3'**

(Reverse) 5' CTCATCCTTGGCTTCCTCAG 3'

*β3-TUBULIN* NM006086.3 (Forward) 5’ GGCCTCTTCTCACAAGTACG 3’

(Reverse) 5’ CCACTCTGACCAAAGATGAAA 3’

*ANKH*  NM054027.4 (Forward) 5’ GAGGTGACAGACATCGTGG 3'

(Reverse) 5' CCTTTAAATCAAGGCCTCTTTCATTAC 3'

*PIT1* NM005415.4 (Forward) 5’ GGAGGGTGTCAAGTGGTCTGAA 3’

(Reverse) 5’ ATCTGCCTTATGGAGGATGAATG 3’

*ENPP1* NM006208.2 (Forward) 5’ AAATATGCAAGCCCTCTTTGT 3’

(Reverse) 5’ TTTAGAAGGTGGTTAAGACTTCCATGA 3’

18S NR003286.2 (Forward) 5’ GGCGTCCCCCAACTTCTTA3’

(Reverse) 5’ GGGCATCACAGACCTGTTATT 3’
