# Supplementary figures and images for: Interleukin 6 promotes an in vitro mineral deposition by stem cells isolated from human exfoliated deciduous teeth
Source: R Soc Open Sci. 2018 Oct 31;5(10):180864. doi: 10.1098/rsos.180864 (PMC6227976; doi:10.1098/rsos.180864)

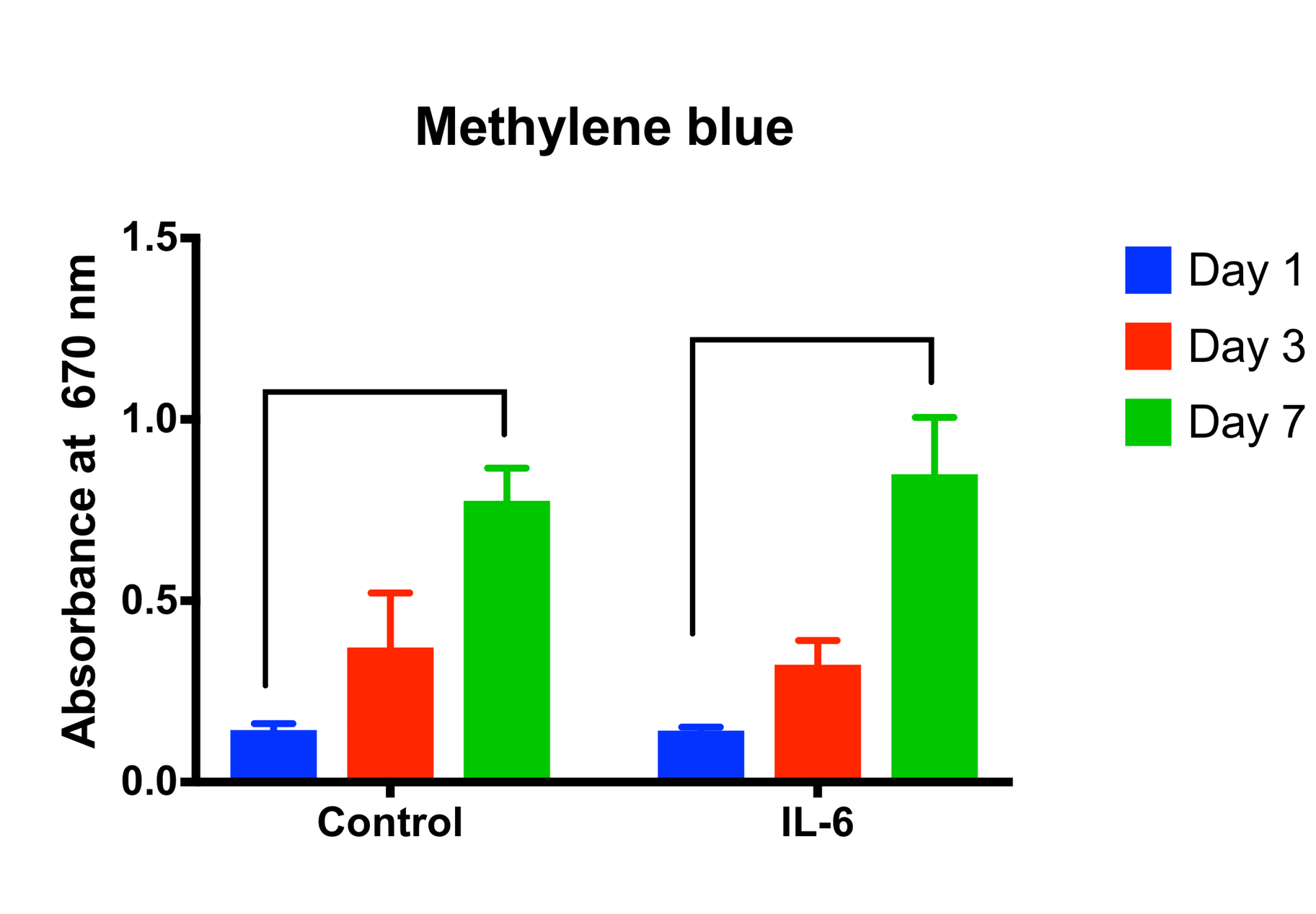

Supplement: Supplementary Figure 1 [file rsos180864supp2.tiff]

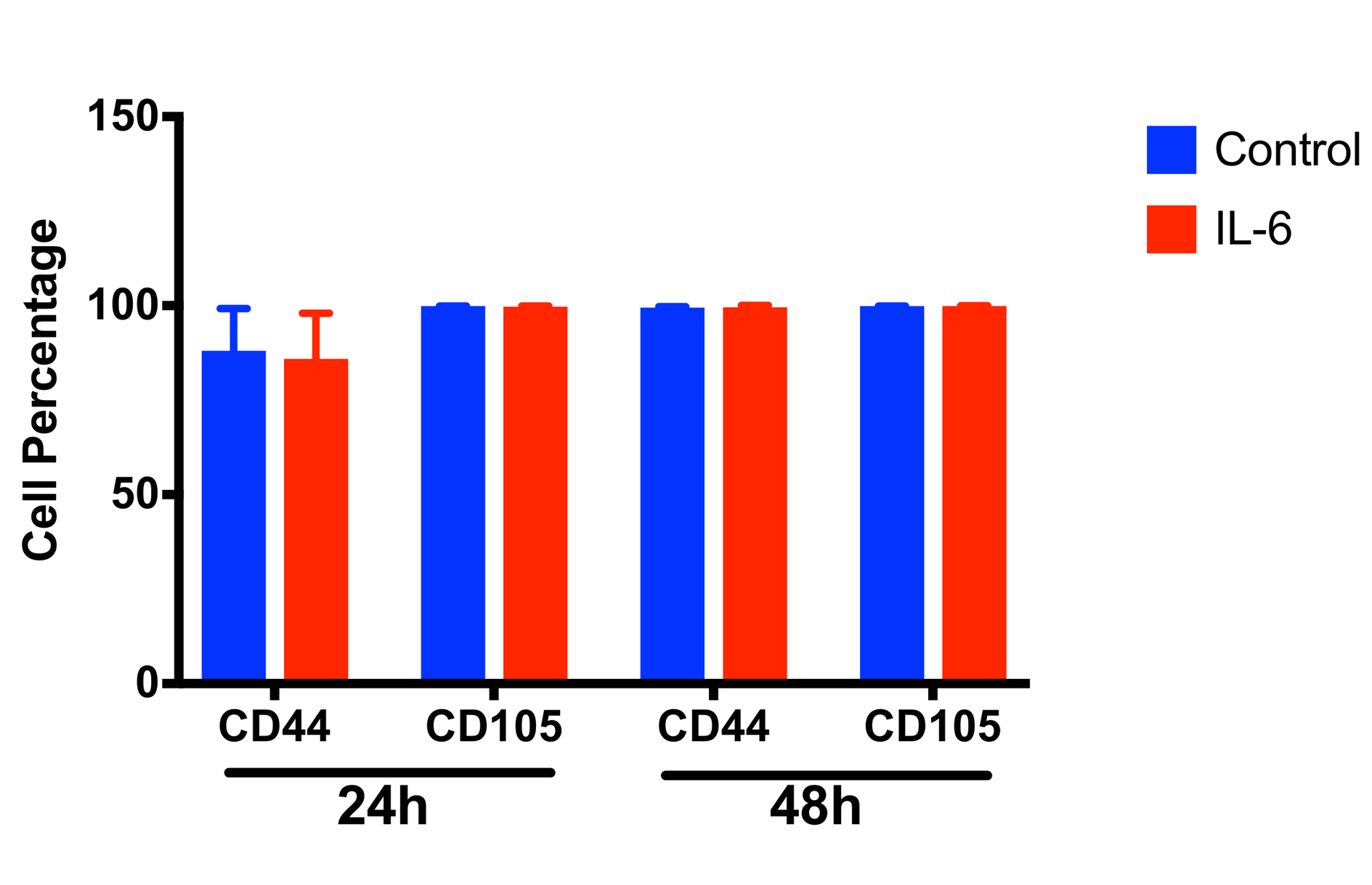

Supplement: Supplementary Figure 2 [file rsos180864supp3.tiff]
